# Fault Feature Analysis of Gear Tooth Spalling Based on Dynamic Simulation and Experiments

**DOI:** 10.3390/ma14206053

**Published:** 2021-10-13

**Authors:** Zhiguo Wan, Jie Zheng, Jie Li, Zhenfeng Man

**Affiliations:** 1 School of Mechanical Engineering, Xi’an Shiyou University, Xi’an 710065, China; zhengjie@xsyu.edu.cn (J.Z.); manzhenfeng2019@163.com (Z.M.); 2 School of Mechanical Engineering, Xi’an Jiaotong University, Xi’an 710049, China; lijie1@stu.xjtu.edu.cn

**Keywords:** gear system, tooth spalling, dynamic simulation, fault feature, health monitoring

## Abstract

Gear dynamics analysis based on time-varying meshing stiffness (TMS) is an important means to understand the gear fault mechanism. Based on Jones bearing theory, a bearing statics model was established and introduced into a gear system. The lateral–torsion coupling vibration model of the gear shaft was built by using a Timoshenko beam element. The lumped parameter method was used to build the dynamic model of a gear pair. The dynamic model of a spur gear system was formed by integrating the component model mentioned above. The influence of rectangular and elliptical spalling on TMS was analyzed by the potential energy method (PEM). The fault feature of tooth spalling was studied by dynamic simulation and verified by experiments. It is found that the gear system will produce a periodic shock response owing to the periodic change of the number of meshing gear teeth. Due to the contact loss and the decrease of TMS, a stronger shock response will be generated when the spalling area is engaged. In the spectrum, some sidebands will appear in the resonance region. The results can provide a theoretical guide for the health monitoring and diagnosis of gear systems.

## 1. Introduction

As a key component to change speed and torque, the gear system plays a significant role in rotating machinery. However, the gear system often operates at high speed, heavy load, and variable operating conditions, which made the gear system often vulnerable to different kinds of damages. Due to the complex structure and long signal transmission path, the gear fault signal is often submerged by strong background noise, so it is difficult to directly observe the gear fault characteristics from the signal collected by the sensor. Therefore, many researchers studied the meshing characteristics of gear systems with cracks, spalling and wear faults through dynamic simulation. At first, the research on gear fault dynamics mainly focused on root crack faults. In those studies, the influence of crack on TMS and dynamic response was mainly analyzed [1,2,3,4]. The results show that the influence of tooth crack on TMS is the main factor to change the vibration characteristics of a gear system. In order to improve the solution accuracy of TMS, an improved method [5] that considered the influences of the tooth number on the gear base circle was proposed and extended to analyze a helical gear system with tooth cracks [6]. Some researchers considered more factors besides TMS in order to make the model more accurate, such as extended tooth contact [7], tooth shape parameters [8], and more degrees of freedom (DOF) [9]. In recent years, the vibration response mechanism of tooth spalling, pitting, and wear has been paid more and more attention. Khaldoon et al. [10] analyzed the influences of tooth face wear on TMS, friction, and the vibration response of the gear system, which shows that the spectral peaks at the meshing frequency components along with their sidebands were the fault characteristics caused by wear. In order to describe the irregularity of tooth pitting, some novel models [11,12] based on probability distribution were proposed, and the influences of tooth pitting on TMS were investigated. Compared with tooth pitting, tooth spalling is a more serious form of tooth surface failure. When the gear spalling fault occurs, the reduction of gear contact length is the main reason for the change of TMS and dynamic response [13]. Simplifying spalling fault as a rectangular or circle shape, Cheng et al. [14], Abouelseoud et al. [15], and Saxena et al. [16] analytically studied the effects of a single pit or spalling on the TMS. Liang et al. [17] investigated the influences of multiple spalling on the TMS, and the spalling was modeled as a circle shape. In order to simulate the irregularity of the spalling shape, a new method based on discretization of the gear tooth profile was proposed to analyze the TMS of a spur gear with a spalling defect [18]. In terms of the influences of spalling fault on TMS, the above researchers have done important work. Unfortunately, they did not do further dynamic analysis. Aiming at dynamic simulation of gear spalling fault, Huangfu et al. [19] utilized the loaded tooth contact analysis method to evaluate the meshing characteristics of spalled gear pairs. The results show that the simulated fault features agree well with that obtained from the experiment.

According to the above, the research on tooth spalling gradually led to more attention. Although some works [11,12,13,14,15,16,17,18] have been done on TMS of gear teeth with spalling or pitting, the research on vibration characteristics caused by spalling faults is still relatively few. In addition, although the bearing system is a key part of gear systems, the bearing was usually simplified to a simple spring system [1,2,3,4,5,6,7,8,19]; few references consider an accurate modeling of bearings. Therefore, a dynamic model considering a bearing system was established to analyze the fault feature of tooth spalling, and the proposed model was verified by experiments. Moreover, the experimental signals were processed by the second-generation wavelet analysis, and the shock response induced by the alternate meshing of single and double gear teeth was found, which is a phenomenon that has hardly been found in other research. The structure of this paper is as follows. In Section 2, the influences of rectangular and elliptical spalling on TMS were studied by the PEM. In Section 3, a gear dynamic model of a spur gear system based on the Jones bearing model, Timoshenko beam elements, and a nonlinear gear pair system model was established. In Section 4, the fault features of tooth spalling were simulated and discussed by dynamic simulation and experiments. Conclusions are given in Section 5.

## 2. The Influence of Tooth Spalling Fault on TMS

The TMS is a major dynamic excitation of a gear system. When tooth fault occurs, the change of TMS is a key reason for the change of system response. At present, most researchers usually use the PEM to calculate the TMS of a gear system.

### 2.1. The Basic Principle of Calculating the TMS by the PEM

**Figure 1:**
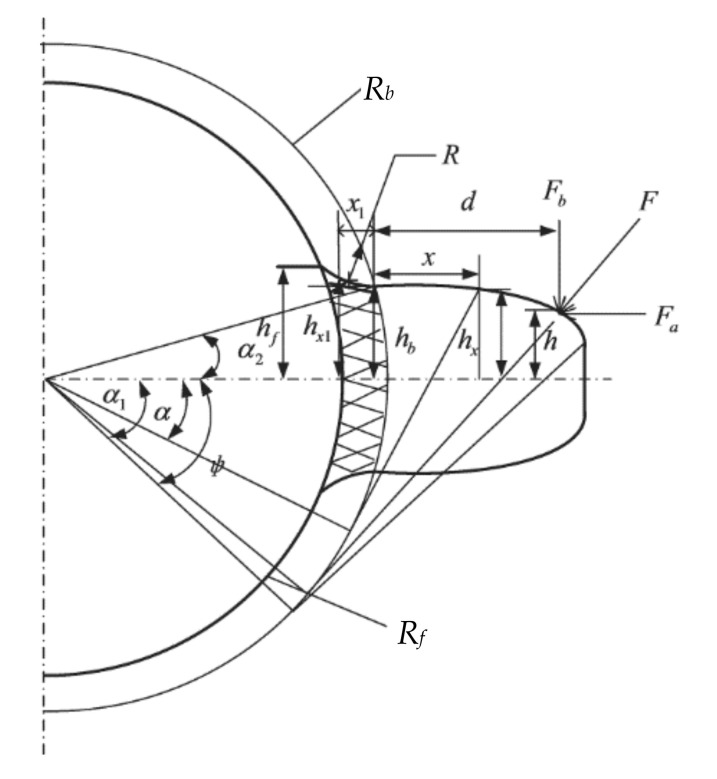
The variable section cantilever beam model of a gear.

As shown in Figure 1, when the TMS is analyzed by the PEM, the tooth is simplified to a variable section cantilever beam model, and the two contact gear teeth bodies can be approximated by two cylinders. The PEM [5,6] assumes that the deformation energy of a meshing gear tooth includes four parts: Hertz contact energy *U*_h_, radial compression deformation energy *U*_a_, bending potential energy *U*_b_, and shear deformation energy *U*_s_. The Hertz contact stiffness *k*_h_, radial compression stiffness *k*_a_, bending stiffness *k*_b_, and shear stiffness *k*_s_ can be calculated by these four kinds of potential energy. In addition to the tooth deformation, the fillet-foundation deflection also has an important effect on the TMS [1,20], and the corresponding stiffness can be expressed as *k*_f_. According to the principle of elasticity and material mechanics, the relationship between stiffness and deformation energy can be expressed as

(1) $$U_{h}=\frac{F^{2}}{2k_{h}}$$

(2) $$U_{b}=\frac{F^{2}}{2k_{b}}\;=\int_{0}^{d}\frac{{\left[F_{b}(d-x)-F_{a}h\right]}^{2}}{2EI_{x}}dx\;+\int_{0}^{R_{b}-R_{f}}\frac{{\left[F_{b}(d+x_{1})-F_{a}h\right]}^{2}}{2EI_{x_{1}}}dx_{1}$$

(3) $$U_{s}=\frac{F^{2}}{2k_{s}}=\int_{0}^{d}\frac{1.2F_{b}{}^{2}}{2GA_{x}}dx+\int_{0}^{R_{b}-R_{f}}\frac{1.2F_{b}{}^{2}}{2GA_{x}}dx$$

(4) $$U_{a}=\frac{F^{2}}{2k_{a}}=\int_{0}^{d}\frac{F_{a}{}^{2}}{2EA_{x}}dx+\int_{0}^{R_{b}-R_{f}}\frac{F_{a}{}^{2}}{2EA_{x}}dx$$

where *F* is the meshing force, *F*_a_ is the radial component of *F*, *F*_b_ is the tangential component of *F*, *E* and *G* are the Young’s modulus and shear modulus, *d* represents the horizontal distance from the meshing position to the base circle, *A_x_* and *I_x_* are the area and area moment of inertia; *h* represents the distance from the meshing position to the symmetry line of the gear teeth; *R*_b_ and *R*_f_ are the base circle radius and root circle radius, respectively.

The TMS of a gear pair can be equivalent to the above stiffness in series, which can be expressed as

(5) $$k=\;\left\{\begin{array}{l} \;\;\;\frac{1}{\frac{1}{k_{h}}+\frac{1}{k_{b1}}+\frac{1}{k_{s1}}+\frac{1}{k_{f1}}+\frac{1}{k_{a1}}+\frac{1}{k_{b2}}+\frac{1}{k_{s2}}+\frac{1}{k_{a2}}+\frac{1}{k_{f2}}}\;\;\;\;\;\;\;\;\;\mathrm{Single}\;\mathrm{tooth}\;\mathrm{meshing}\;\\ \;\;\sum_{i=1}^{2}\frac{1}{\frac{1}{k_{h,i}}+\frac{1}{k_{b1,i}}+\frac{1}{k_{s1,i}}+\frac{1}{k_{f1,i}}+\frac{1}{k_{a1,i}}+\frac{1}{k_{b2,i}}+\frac{1}{k_{s2,i}}+\frac{1}{k_{a2,i}}+\frac{1}{k_{f2,i}}}\;\;\mathrm{Double}\;\mathrm{teeth}\;\mathrm{meshing}\;\;\; \end{array}\right.$$

where subscripts “1” and “2” represent the pinion and gear, respectively.

### 2.2. The Influence of Tooth Spalling Fault on TMS

**Figure 2:**
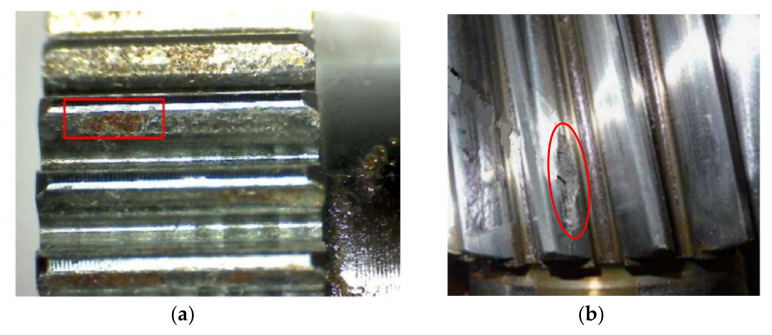
Actual tooth spalling: (**a**) Rectangular spalling, (**b**) Elliptic spalling.

**Figure 3:**
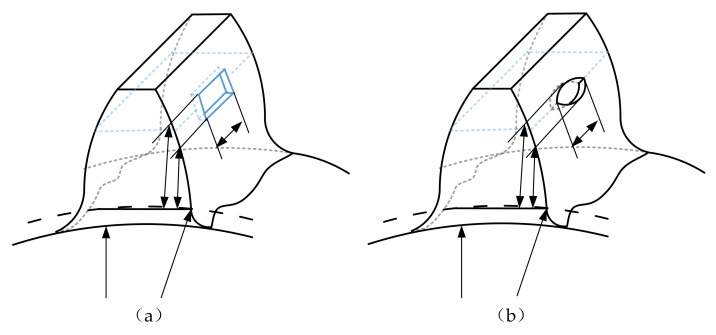
The model of tooth spalling: (**a**) Rectangular spalling, (**b**) Elliptic spalling.

Near the pitch line, the sliding directions of the driving gear and driven gear are opposite, forming a pulsating cyclic load and resulting in a tooth surface spalling fault. Some actual pictures of tooth spalling in engineering are illustrated in Figure 2. According to the shape of tooth spalling in Figure 2, a simplified model as shown in Figure 3 was obtained. When the spalling fault occurs, the effective contact tooth width, the cross-sectional area, and the moment of inertia at the spalling position will change, thus changing the Hertz contact stiffness, radial compression stiffness, bending stiffness, and shear stiffness of the spalling area.

As shown in Figure 3, in the range of tooth width corresponding to the spalling area, the effective cross-sectional area and moment of inertia at distance *x* from the base circle can be expressed as follows

(6) $$I_{x}=\left\{\begin{array}{c} \;\;\;\frac{1}{12}{\left(2h_{x}\right)}^{3}\Delta L\;\;x\leq x_{s}\;\mathrm{or}\;x>x_{e}\\ \frac{1}{12}{\left(2h_{x}-\Delta h\right)}^{3}\Delta L\;\;x_{s}<x<x_{e} \end{array}\right.,\;\;A_{x}=\left\{\begin{array}{c} \;\left(2h_{x}\right)\Delta L\;\;x\leq x_{s}\;\mathrm{or}\;x>x_{e}\\ (2h_{x}-\Delta h)\Delta L\;\;x_{s}<\;x<x_{e}\; \end{array}\right.$$

where Δ*L* is the tooth width corresponding to the spalling area, Δ*h* is the depth of the spalling area, *x_s_* and *x_e_* are the distance from the upper and lower boundaries of the spalling area to the tooth base circle.

Incorporating Equation (6) into Equations (1)–(5), the calculation formula of meshing stiffness of gear with spalling fault can be obtained. For instance, the bending stiffness can be expressed as

(7) $$\frac{1}{k_{b}}\;=\left\{\begin{array}{c} \;\int_{0}^{d}\frac{M^{2}}{EI_{x}}\;\;dx+\int_{0}^{R_{b}-R_{f}}\frac{M_{1}{}^{2}}{EI_{x_{1}}}dx_{1}\;\;\;\;\;\;\;\;\;\;\;\;\;\;\;\;\;\;\;\;\;\;\;\;\;d\leq x_{s}\\ \;\int_{0}^{x_{s}}\frac{M^{2}}{EI_{x}}dx\;+\int_{x_{s}}^{d}\frac{M^{2}}{EI_{x}}dx\;+\int_{0}^{R_{b}-R_{f}}\frac{M_{1}{}^{2}}{EI_{x_{1}}}dx_{1}\;\;\;\;\;\;\;\;\;\;\;x_{s}<\;d\;<\;x_{d}\\ \int_{0}^{x_{s}}\frac{M^{2}}{EI_{x}}\;dx+\int_{x_{s}}^{x_{e}}\frac{M^{2}}{EI_{x}}\;dx+\int_{x_{e}}^{d}\frac{M^{2}}{EI_{x}}dx\;+\int_{0}^{R_{b}-R_{f}}\frac{M_{1}{}^{2}}{EI_{x_{1}}}dx_{1}\;\;\;d>x_{d} \end{array}\right.$$

where $M=F_{b}(d-x)-F_{a}h,\;M_{1}=F_{b}(d+x_{1})-F_{a}h$; the meaning of other parameters is the same as in Formulas (1)–(5) and Figure 1.

Due to the decrease of the contact length in the spalling region, the calculation of Hertz contact stiffness will be replaced by the following equation.

(8) $$k_{h}=\frac{\pi E(L-\Delta L)}{4(1-v^{2})}$$

where *v* is Poisson’s ratio.

**Figure 4:**
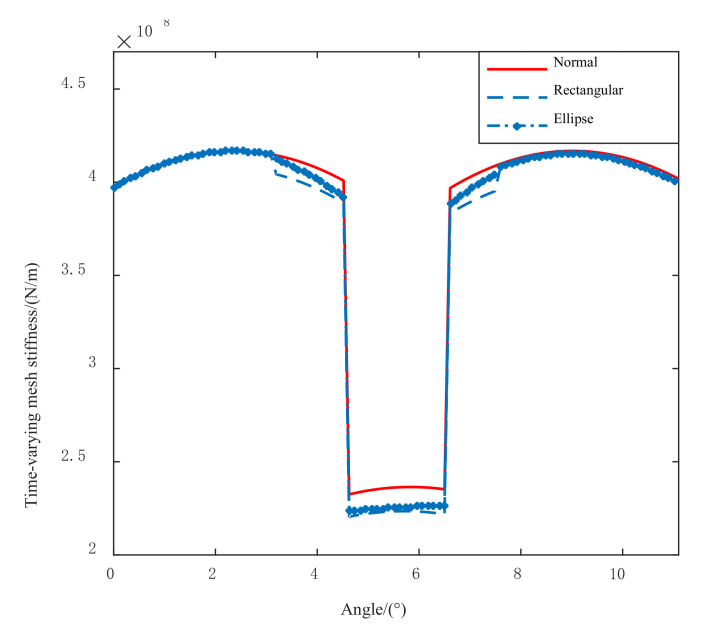
The TMS of tooth spalling.

It is assumed that the length and width of tooth spalling are 5 mm and 3 mm, respectively. The TMS of the spalling fault shown in Figure 4 can be obtained through the above equations. It can be found that the decrease of TMS appears in the spalling area, which is essentially different from the change of TMS caused by the tooth root crack. The influence of tooth crack on TMS can be referred to Ref [3]. Compared with elliptical spalling, the TMS changes abruptly when the rectangular spalling area starts to mesh or moves out of engagement. For elliptical spalling, the TMS changes gradually because of the gradual change of cross-sectional area, moment of inertia, and contact length.

## 3. The Nonlinear Dynamic Model of Gear System

### 3.1. The Dynamic Model of Gear Shaft

**Figure 5:**
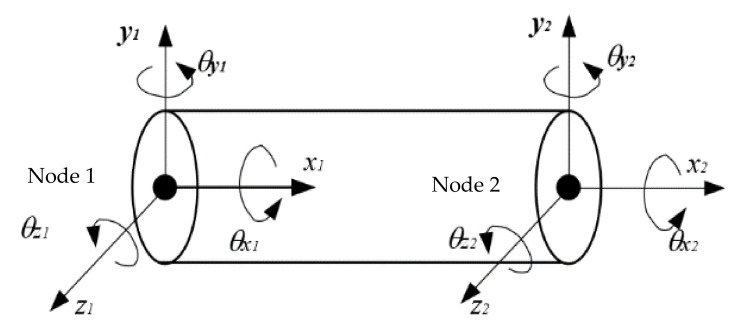
Timoshenko beam element.

The transmission shaft of a gear system can be modeled by using a Timoshenko beam element model with two nodes and six DOF. As shown in Figure 5, each node of the Timoshenko beam element includes three translational DOF, x, y and z, three rotational DOF, $\theta_{x}$, $\theta_{y}$ and $\theta_{z}$. it is assumed that the element’s generalized coordinate vector is $q_{e}^{}$, the dynamic equation of the transmission shaft was obtained from the Lagrange equation

(9) $$M_{e}^{}{\ddot{q}}_{e}^{}+(C_{e}-\omega G_{e}^{}){\dot{q}}_{e}^{}+K_{e}^{}q_{e}^{}=Q_{e}^{}$$

where $M_{e}$ is the mass matrix, $K_{e}$ is the stiffness matrix, $G_{e}$ is the gyro matrix, $Q_{{}^{e}}$ is the generalized force vector of the elastic shaft, ω is the angular velocity, $C_{e}$ is the damping matrix which is commonly calculated by Rayleigh damping.

### 3.2. Nonlinear Bearing Model

**Figure 6:**
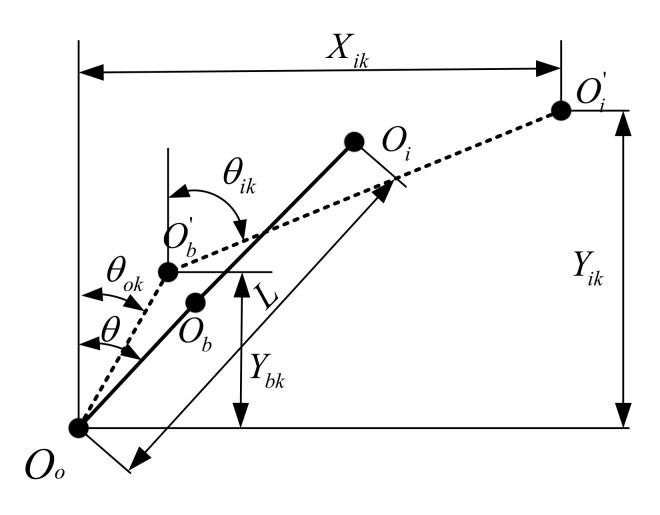
Positions relationship of ball center, inner and outer ring curvature center.

When the bearing runs at high speed, the relative position of the outer ring, inner ring and the ball center will change under the external load. The geometric relationship between the internal elements of the bearing after deformation is shown in Figure 6. In this figure, $O_{o}$ is the curvature center of the bearing outer ring, $O_{i}$ is the curvature center of the bearing inner ring, $O_{b}$ is the spherical center position of the rolling element, $O_{i}^{\prime }$ is the curvature center of the bearing inner ring raceway after loading, $O_{b}^{\prime }$ is the spherical center position of the of the rolling body after loading, $\theta_{ok}$ and $\theta_{ik}$ are the contact angles of the rolling element with the inner and outer rings after loading, respectively, $X_{ik}$ and $Y_{ik}$ are the axial and radial distances between the curvature centers of the inner and outer rings.

Considering the centrifugal force, inertial force, friction and other factors, a static analysis model of the rolling element under any load can be established [21]. The ball-inner raceway contact deformation $\delta_{ik}$ and ball-outer raceway contact deformation $\delta_{ok}$ can be gotten by the Newton-Raphson method. Next, the inner ring contact force $Q_{ik}$ and outer ring contact force $Q_{ok}$ are deduced by Hertz contact theory for spherical contact.

The nonlinear contact force and torque acting on the inner ring can be obtained by adding the contact force between all balls and the bearing inner ring and balancing with external force on the bearing inner ring

(10) $$\left\{\begin{array}{c} F_{xi}=\sum_{k=1}^{N}\left(Q_{ik}\sin\theta_{ik}+\frac{M_{gk}}{D}\cos\theta_{ik}\right)\\ F_{yi}=\sum_{k=1}^{N}\left(Q_{ik}\cos\theta_{ik}-\frac{M_{gk}}{D}\sin\theta_{ik}\right)\cos\varphi_{k}\\ F_{zi}=\sum_{k=1}^{N}\left(Q_{ik}\cos\theta_{ik}-\frac{M_{gk}}{D}\sin\theta_{ik}\right)\sin\varphi_{k}\\ M_{yi}=+\sum_{k=1}^{N}\left\{r_{ic}\left(Q_{ik}\sin\theta_{ik}+\frac{M_{gk}}{D}\cos\theta_{ik}\right)-f_{i}M_{gk}\right\}\sin\varphi_{k}\\ M_{zi}=-\sum_{k=1}^{N}\left\{r_{ic}\left(Q_{ik}\sin\theta_{ik}+\frac{M_{gk}}{D}\cos\theta_{ik}\right)-f_{i}M_{gk}\right\}\cos\varphi_{k} \end{array}\right.$$

where $r_{oc}=D_{m}/2-(f_{o}-0.5)D\cos\theta$, $D_{m}$ and $M_{gk}$ are pitch diameter and gyroscopic torque of the ball, $f_{i}$ and $f_{o}$ are the curvature radius of the inner ring and outer ring, *D* is the rolling ball diameter. The detailed derivation of this formula was given in Ref [21].

Taking the derivative of the force to displacement, the stiffness matrix of the bearing system was expressed as

(11) $$K_{b}=\frac{\partial F}{\partial \delta }$$

where $F=[F_{xi},F_{yi},F_{zi},M_{xi},M_{zi}{]}^{T}$ is the force vector, $\delta =[\delta_{xi},\delta_{yi},\delta_{zi},\delta_{xi},\gamma_{yi},\gamma_{zi}{]}^{T}$ is the displacement vector of bearing inner ring.

### 3.3. Nonlinear Dynamic Model of Gear Pair

**Figure 7:**
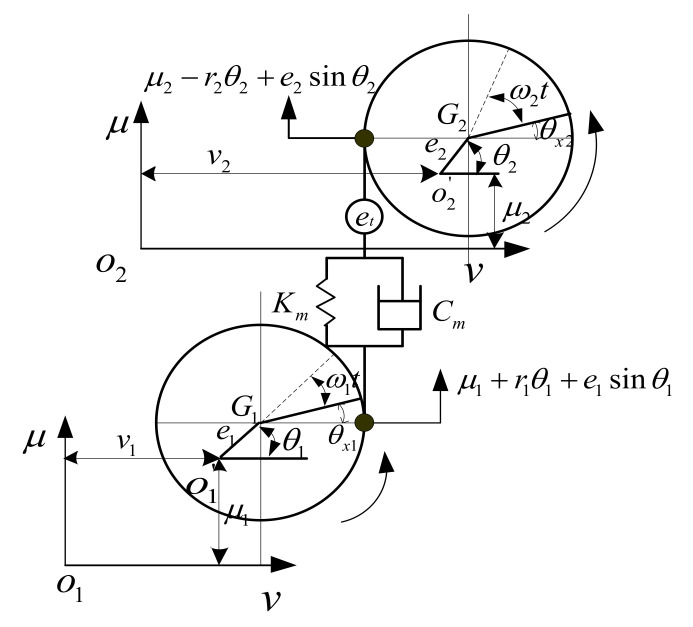
The gear pair dynamic model.

The gear pair dynamic model is generally established by the lumped parameter method. A dynamic model with multiple DOF is illustrated in Figure 7. Some factors such as the geometric transmission error, geometric eccentricity, and gyroscopic effects were taken into account. In this model, u and v represent the horizontal and vertical translational DOF, respectively. $\theta_{u}$, $\theta_{v}$ and $\theta_{x}$ represent the rotational DOF in three directions, respectively; $O_{1}^{}$ and $O_{2}$ are the center of the pinion and gear when the gear system are stationary; $O_{1}^{\prime }$ and $O_{2}^{\prime }$ represent the center of the pinion and gear when the gear system are rotating; *G*_1_ and *G*_2_ represent the geometric center of the pinion and gear, respectively.

According to the principle of force and torque balance, the dynamic equation of the gear pair system can be obtained

(12) $$M_{1}{\ddot{\mu }}_{1}+K_{m}x+C_{m}\dot{x}=M_{1}e_{1}\omega_{1}{}^{2}\sin\theta_{1}+K_{m}e_{s}+C_{m}{\dot{e}}_{s}$$

(13) $$M_{1}{\ddot{v}}_{1}=M_{1}e_{1}\omega_{1}{}^{2}\cos\theta_{1}$$

(14) $$M_{2}{\ddot{\mu }}_{2}-K_{m}x-C_{m}\dot{x}=M_{2}e_{2}\omega_{2}{}^{2}\sin\theta_{2}-(K_{m}e_{s}+C_{m}{\dot{e}}_{s})$$

(15) $$M_{2}{\ddot{v}}_{2}=M_{2}e_{2}\omega_{2}{}^{2}\cos\theta_{2}$$

(16) $$J_{d1}{\ddot{\theta }}_{v1}+J_{p1}\omega_{1}{\dot{\theta }}_{\mu 1}=0$$

(17) $$J_{d1}{\dddot{\theta }}_{\mu 1}-J_{p1}\omega_{1}{\dot{\theta }}_{v1}=0$$

(18) $$J_{p1}{\ddot{\theta }}_{1}+K_{m}r_{1}x+C_{m}r_{1}\dot{x}=[-K_{m}x-C_{m}\dot{x}+K_{m}e_{s}+C_{m}{\dot{e}}_{s}]e_{1}\cos\theta_{1}+[K_{m}e+C_{m}\dot{e}]r_{1}$$

(19) $$J_{d2}{\ddot{\theta }}_{v2}+J_{p2}\omega_{2}{\dot{\theta }}_{\mu 2}=0$$

(20) $$J_{d2}{\ddot{\theta }}_{u2}-J_{p2}\omega_{2}{\dot{\theta }}_{v2}=0$$

(21) $$J_{p2}{\ddot{\theta }}_{2}+K_{m}r_{2}x+C_{m}r_{2}\dot{x}=[-K_{m}x-C_{m}\dot{x}+K_{m}e_{s}+C_{m}{\dot{e}}_{s}^{}]e_{2}\cos\theta_{2}+[K_{m}e+C_{m}\dot{e}]r_{2}$$

where $x=\mu_{1}+r_{1}\theta_{1}-\mu_{2}+r_{2}\theta_{2}$, $e_{s}=e_{2}\sin\theta_{2}-e_{1}\sin\theta_{1}+e_{t}$, M is the mass; $J_{d}$ and $J_{p}$ are the transverse and polar moments of inertia, respectively; $K_{m}$ and $C_{m}$ represent the TMS and meshing damping of the gear pair system; r is the base circle radius; $e_{t}$ is the static transmission error; e represents the geometric eccentricity; ω represents the angular rotation speed; the subscripts “1” and “2” indicate the pinion and the gear, respectively.

### 3.4. The Dynamic Model of Gear System

**Figure 8:**
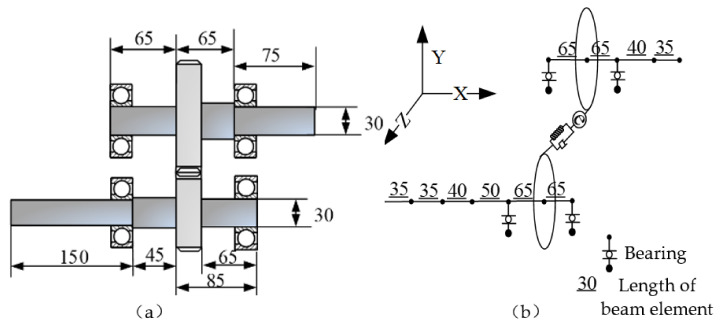
A gear system: (**a**) Structure diagram, (**b**) Dynamic model.

Figure 8 is the structure diagram and dynamic model diagram of a gear transmission system. In the dynamic model shown in Figure 8b, the transmission shaft is established by using the beam element described in Section 3.1, the Jones bearing theory described in Section 3.2 is used for bearing modeling, and the theory described in Section 3.3 is used for gear pair modeling. After getting the dynamic equations of each substructure, according to the node where the gear and bearing are located, the mass, stiffness, gyro matrix of gear pair system, and stiffness matrix of bearing are superimposed on the corresponding node of elastic shaft element; then, the dynamic equation of the whole gear system can be obtained

(22) $$M\ddot{q}+[G+C]\dot{q}+Kq=F$$

where M, C, G and K are the mass, damping, gyro and damping matrix of the gear transmission system, respectively; F is the generalized force.

## 4. Fault Feature Analysis of Tooth Spalling Based on Dynamic Simulation and Experiments

### Dynamic Simulation and Experiment Verification of Gear System without Defect

**Figure 9:**
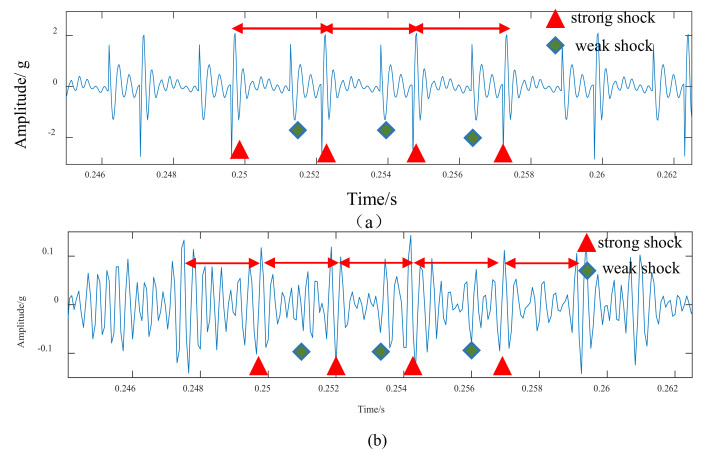
Time domain signal of normal system: (**a**) Simulation, (**b**) Experimental.

**Figure 10:**
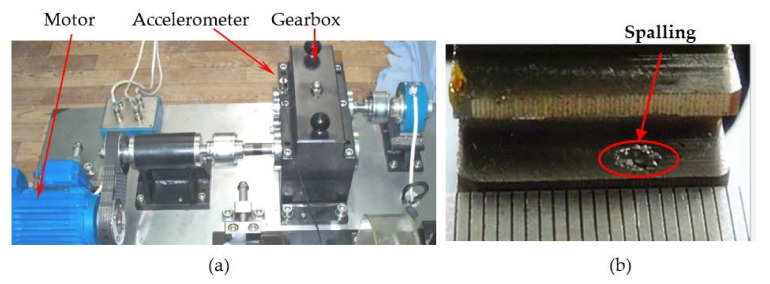
(**a**) A gearbox test, (**b**) Tooth spalling.

**Figure 11:**
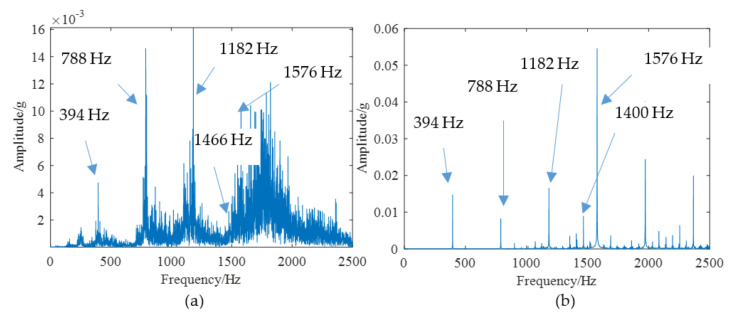
The spectrum of normal system: (**a**) Experimental, (**b**) Simulation.

According to the above modeling theory and the parameters shown in Table 1, the gear system dynamics equation is established. The time domain simulation signal shown in Figure 9a can be obtained by the Newmark-β numerical solution method. It is worth noting that the unit “g” represents the acceleration of gravity in all the time domain signals. As shown in Figure 10, a test bench was built for verifying the simulation model. The parameters of this test bench are shown in Table 1. The vibration acceleration signals of the gear system can be collected by an accelerometer sensor positioned on the top of the gearbox casing. The length and width of the spalling shown in Figure 10b are about 4 mm length and 3 mm, respectively. The sampling frequency of the data acquisition system was set at 12.8 KHz. The rotation frequency of the pinion shaft *f_p_*_1_ was 7.2 Hz, the rotational frequency of the driving gear *f_p_*_2_ is about 5.26 Hz, and the meshing frequency was about 394 Hz. A second generation wavelet method [22] is adopted to decompose the original signal due to its large background noise. Figure 9b shows the fourth subspace of the original signal. According to Figure 9, it can be found that some shock response will be produced in the teeth engagement process. This is mainly due to the abrupt change of TMS caused by the change of the number of meshing teeth. Each group is composed of two alternating periodic shock responses with different amplitude—a strong shock and a weak shock. This phenomenon is clearly captured by simulation and experiment signals. However, since the signal in Figure 9b is the subband signal after the second-generation wavelet decomposition, the amplitude has a certain attenuation compared with the original signal. Therefore, there is a great difference in amplitude between the two signals in Figure 9. The spectrum of the simulation signal and experiment signal are obtained by Fourier transform, and the spectrum within 2500 Hz is shown in Figure 11, which shows that the frequency components of the normal signal are mainly the meshing frequency and its frequency multiplication component.

**Figure 12:**
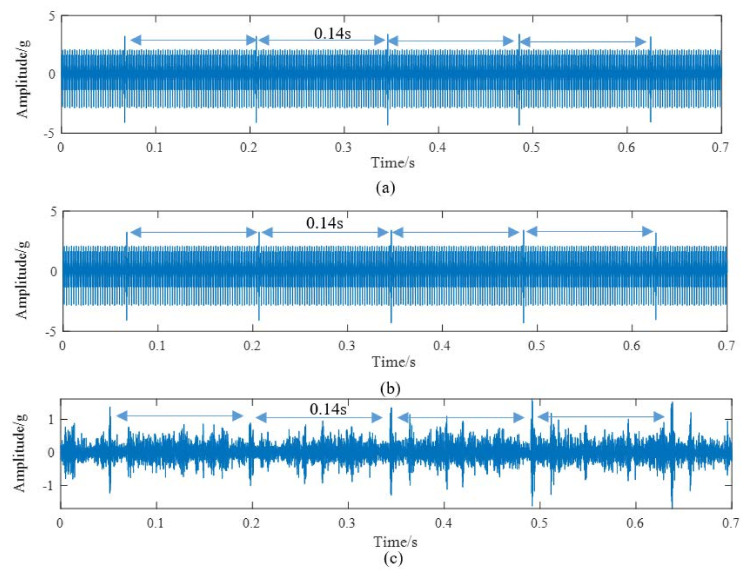
Time domain signals of tooth spalling: (**a**) Simulation signal of rectangular spalling, (**b**) Simulation signal of elliptical spalling, (**c**) Experimental signal of elliptical spalling.

**Figure 13:**
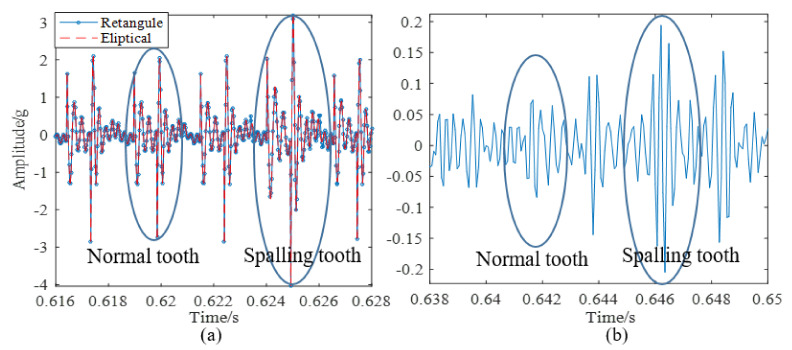
Local amplification of time domain signals: (**a**) Simulation signal, (**b**) Experimental signal, 4.2 dynamic simulation, and experimental verification of tooth spalling fault.

**Figure 14:**
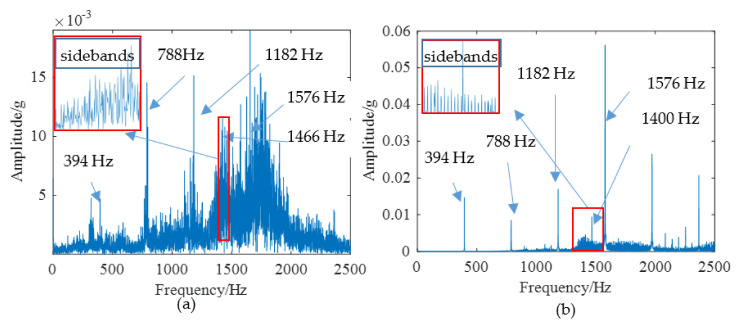
The spectrum of spalling fault signal: (**a**) Experimental, (**b**) Simulation.

The spalling fault shown in Figure 3 is introduced to the proposed model, the TMS shown in Figure 4 is brought into Equation (22), and the dynamic simulation is carried out with the same parameters and methods. The time-domain simulation waveform of rectangular spalling and elliptical spalling fault is obtained and shown in Figure 12a,b. More common elliptical spalling faults in engineering are processed (as shown in Figure 10b), and the experiment signal is shown in Figure 12c. It can be found that the spalling fault will produce a series of periodic vibrations and impact responses whether the tooth spalling shape is rectangular or elliptical. The impulse period caused by the spalling fault is the rotational period of the defected gear. Figure 13 is the local amplification of time-domain signals. When the normal teeth of the faulty gear engage, the vibration signal is the same as that of the normal gear shown in Figure 9. When the spalling tooth comes in contact, the impulse amplitude will increase. This shows that the sudden change of TMS caused by gear spalling will stimulate the shock response of the system. For every rotation of the gear, the spalling teeth mesh once, which will produce a shock response with larger amplitude. Fourier transform was applied to the simulation; the experiment signal is shown in Figure 12b,c, and the spectrum is shown in Figure 14. Compared with Figure 11, Figure 14 shows that there are some sidebands with significant amplitudes due to the short-period nature of the shock. Those sidebands mainly occur in the resonance region and expand to higher frequencies. These characteristic are also in good agreement with other research conclusions [23].

## 5. Conclusions

In order to provide a theoretical guide for the health monitoring and diagnosis of a gear system, based on Jones bearing theory, Timoshenko beam element modeling theory, and nonlinear gear pair system modeling theory, a gear dynamic simulation model was proposed. The TMS of a gear tooth with a spalling fault was deduced based on the PEM. The influences of tooth spalling on the response characteristics were investigated through dynamic simulation and experiments. The main conclusions are summarized as follows:

(1) An analytical calculation formula was proposed to analyze the influence of gear spalling fault on TMS. The results show that spalling faults can cause a local reduction of TMS.

(2) Although many researchers show that the shock response will be generated due to the alternating engagement of single and double gear teeth, few experimental signals have been used to verify this phenomenon. In this paper, the second-generation wavelet analysis was used to process the experimental signals, and the shock response induced by the alternate meshing of single and double gear teeth was found, which proves the correctness of the gear dynamics analysis model based on the TMS.

(3) When tooth spalling fault occurs, the decrease of TMS mainly occurs in the spalling area, which is essentially different from the reduction of the TMS due to tooth root cracks. Due to the contact loss and the decrease of TMS, a shock response with a larger amplitude will be generated when the spalling area engages. In the frequency domain of the vibration signal, some sidebands will appear in the resonance area.

## Figures and Tables

**Table 1:** Parameters of a gear system.

	Parameters	Pinion	Gear
Gear	Teeth number	55	75
	Module (mm)	2	2
	Pressure angle (°)	20	20
	Tooth width (mm)	20	20
	Moment of inertia (kg.m^2^)	0.003	0.01
	Eccentricity (um)	20	20
Shaft	Radius (mm)	15	

## Data Availability

The data presented in this study are available on request from the corresponding author.
